# 2023 Update on Sepsis and Septic Shock in Adult Patients: Management in the Emergency Department

**DOI:** 10.3390/jcm12093188

**Published:** 2023-04-28

**Authors:** Matteo Guarino, Benedetta Perna, Alice Eleonora Cesaro, Martina Maritati, Michele Domenico Spampinato, Carlo Contini, Roberto De Giorgio

**Affiliations:** 1 Department of Translational Medicine, St. Anna University Hospital of Ferrara, University of Ferrara, 44121 Ferrara, Italy; 2 Infectious and Dermatology Diseases, St. Anna University Hospital of Ferrara, University of Ferrara, 44121 Ferrara, Italy

**Keywords:** emergency department, in-hospital mortality, management, sepsis, septic shock

## Abstract

Background: Sepsis/septic shock is a life-threatening and time-dependent condition that requires timely management to reduce mortality. This review aims to update physicians with regard to the main pillars of treatment for this insidious condition. Methods: PubMed, Scopus, and EMBASE were searched from inception with special attention paid to November 2021–January 2023. Results: The management of sepsis/septic shock is challenging and involves different pathophysiological aspects, encompassing empirical antimicrobial treatment (which is promptly administered after microbial tests), fluid (crystalloids) replacement (to be established according to fluid tolerance and fluid responsiveness), and vasoactive agents (e.g., norepinephrine (NE)), which are employed to maintain mean arterial pressure above 65 mmHg and reduce the risk of fluid overload. In cases of refractory shock, vasopressin (rather than epinephrine) should be combined with NE to reach an acceptable level of pressure control. If mechanical ventilation is indicated, the tidal volume should be reduced from 10 to 6 mL/kg. Heparin is administered to prevent venous thromboembolism, and glycemic control is recommended. The efficacy of other treatments (e.g., proton-pump inhibitors, sodium bicarbonate, etc.) is largely debated, and such treatments might be used on a case-to-case basis. Conclusions: The management of sepsis/septic shock has significantly progressed in the last few years. Improving knowledge of the main therapeutic cornerstones of this challenging condition is crucial to achieve better patient outcomes.

## 1. Introduction

Sepsis is defined as a life-threatening organ dysfunction caused by a dysregulated host response to infection. Septic shock should be considered a subset of sepsis in which underlying circulatory, cellular, and metabolic abnormalities contribute to a greater risk of mortality than that posed by sepsis alone [1]. Both sepsis and septic shock represent a major growing global burden and a challenge for emergency physicians because of their increasing incidence and great pathophysiological, molecular, genetic, and clinical complexity [1,2,3]. The incidence of sepsis and septic shock has continuously increased since the first consensus definition (Sepsis-1) in 1991, reaching around 49 million cases of sepsis and 11 million sepsis-related deaths worldwide in 2017 [4,5]. These data led the World Health Organization (WHO) to declare sepsis a global health priority [5]. This alarming increase in incidence can be attributed to different factors: (i) the advanced average age among patients, especially in western countries; (ii) the increased number of invasive procedures; (iii) the wide usage of immunosuppressive drugs and chemotherapy; and (iv) antibiotic resistance [6]. Despite significant advancements in therapeutic management, septic patients have a high risk of in-hospital mortality (IHM), accounting for approximately 20% of all-cause deaths globally, rendering this combined ailment one of the highest-mortality conditions encountered in the emergency department (ED) [5,7,8,9].

The frequency of identifiable microorganisms in sepsis/septic shock has varied over time, with a current preponderance of Gram-positive bacteria and an increased clinical and epidemiological significance of fungal sepsis. Among the Gram-positive bacteria, the most frequently isolated pathogens are *Staphylococcus aureus* and *Streptococcus pneumoniae*, whereas among the Gram-negative bacteria, those most commonly identified are *Escherichia coli*, *Klebsiella*, and *Pseudomonas* spp. Among the fungal infections associated with the condition, the predominant role is played by *Candida* spp., which can often be identified in immunosuppressed or neoplastic patients undergoing long-term treatment with chemotherapeutic and immunosuppressive drugs [10]. The main sites of infection related to sepsis are the respiratory tract/pulmonary parenchyma (43%); the urinary system (16%); the abdomen (14%); the head, which is associated with a fever of unknown origin (FUO) (14%); and other sites/causes (13%) [6,10].

From a pathogenetic standpoint, sepsis is currently considered the result of several mechanisms that simultaneously involve a wide range of pro- and anti-inflammatory mediators [11]. Furthermore, sepsis-related cellular modifications have recently been defined, and the importance of microcirculation has been emphasized in the progression from sepsis to septic shock [12]. In this context, the endothelium has been identified as the fundamental functional unit in the pathophysiology of sepsis due to its role in the regulation of microcirculation and the modulation of coagulation mechanisms and inflammatory and anti-inflammatory signaling processes [12,13]. The glycocalyx is a component of the endothelial membrane consisting of proteoglycans and glycoproteins [14]. It mediates different functions, such as the construction of a mechanical barrier regulating vascular permeability, the activation of leukocytes and platelet adhesion, and the modulation of the inflammatory/anti-inflammatory response. Damage to the glycocalyx’s morpho-functional integrity (known as “glycocalyx shedding”) can occur due to oxidizing agents, cytokines, exotoxins, and bacterial endotoxins. This event leads to leukocyte diapedesis and increased vascular permeability with the production of oedema, which raises interstitial pressure and worsens tissue perfusion [14].

According to the third international consensus on sepsis and septic shock (Sepsis-3), sepsis should be suspected in patients with infections stemming from any infective source [1]. In these subjects, a quick Sequential Organ Failure Assessment (qSOFA) should be considered, for which a result ≥ 2 indicates patients who are at higher risk of in-hospital death. However, the 2021 guidelines discourage the use of qSOFA as the sole screening tool, recommending the use of the National Early Warning Score (NEWS) or systemic inflammatory response syndrome (SIRS) score instead due to their better sensitivity vs. qSOFA in predicting patient’s outcome [2]. A diagnosis of sepsis is confirmed in the case of a Sequential Organ Failure Assessment (SOFA) score ≥ 2. Septic shock is defined by the need for a vasopressor to maintain a patient’s mean arterial pressure (MAP) ≥ 65 mmHg and serum lactate level ≥ 2 mmol/L [1]. Based on this background, we wrote the present review to provide emergency physicians with a thorough update on the management of sepsis and septic shock, focusing on each pillar of the pharmacological approach to these conditions.

## 2. Search Strategy

PubMed, Scopus, and EMBASE were searched from inception with particular attention to the November 2021 (release date of latest sepsis guidelines)–January 2023 period. The search terms used were “sepsis” OR “septic shock” AND “adult” AND “management” OR “therapy” AND “Emergency Department”. In addition, we expanded our analysis through a manual search of the references of the included studies and previous reviews.

## 3. Main Text

The following paragraphs will detail the main aspects of sepsis/septic shock management.

### 3.1. Antimicrobials

Antimicrobial therapy is the first pillar of sepsis/septic shock treatment. The administration of a prompt, empiric, antimicrobial therapy at the time of sepsis’s identification and after the collection of the appropriate cultures is a crucial step in pharmacological management. Microbiological samples should be assessed as soon as possible on admission to the ED and include blood and fluid or tissue from other sites deemed proper based on a clinical evaluation (e.g., urine or cerebrospinal fluid). Indeed, particularly in cases of septic shock, every hour of delay is associated with a significant increase in mortality [2,15,16]. The choice of empiric antimicrobial therapy based on clinical (i.e., site of infection, previous antibiotic use, immunosuppression, and risk factors for resistant organisms) and epidemiological criteria is fundamental. Initially, regarding septic shock, multidrug antimicrobial regimens with a wide spectrum of activity should be used (e.g., carbapenems and anti-Gram-negative antimicrobials with dual coverage). Dual coverage for Gram-negative organisms might be appropriate in cases of high suspicion for multidrug-resistant organisms (e.g., Pseudomonas aeruginosa or Acinetobacter baumanii). Dual coverage for Gram-positive organisms and methicillin-resistant *Staphylococcus aureus* (MRSA) should be considered for patients with a high risk of infection due to these pathogens [17]. Since efficacy depends on the peak of the antimicrobic blood level and the length of time during which this level remains above the minimum inhibitory concentration (MIC) for the identified pathogen, appropriate drug dosing is crucial. An initial loading dose may be the best strategy to achieve a therapeutic blood level more rapidly, with further dosing based on renal/liver function and consultation with an infectious disease physician [15,16,17]. Furthermore, antimicrobial treatment should be re-evaluated daily with the aim of a correct de-escalation as soon as the results of cultural tests are available [17,18,19,20,21,22,23,24]. The choice of the most appropriate empiric antimicrobial therapy is often challenging; therefore, it might be useful to consider several risk factors for pathogens that most commonly appear as etiological agents of sepsis (Table 1) [25].

However, the urgent need to establish antimicrobial treatment should be carefully pondered in terms of potential harm related to drugs administered to patients without an infection [2,26,27]. Different studies have proposed comparisons between 1 h vs. 3 h bundles with respect to antimicrobial administration [15,26,27,28,29,30,31,32,33,34,35,36,37,38,39,40,41,42,43,44,45,46]. Current guidelines propose the administration of antimicrobials immediately, ideally within 1 h, in patients for whom sepsis is highly suspected with/without shock or when sepsis is possible and shock is detectable. In cases with a low-to-moderate risk of sepsis without signs of shock, the administration of antimicrobials is recommended within 3 h if concern for infection persists and after performing an assessment of infectious vs. non-infectious causes [2].

Procalcitonin (PCT), a peptide precursor of calcitonin, is widely used for differentiating bacterial vs. non-bacterial infections or other inflammatory conditions [47,48,49]. In the last few years, different authors have proposed PCT as a marker to guide physicians in terms of starting antimicrobial treatment in patients with an unclear clinical presentation [50,51,52,53,54]. However, as expressed in SSC guidelines, PCT associated with clinical evaluation was less effective than clinical evaluation alone with respect to deciding when to start antimicrobials [2]. Recently, presepsin (PSP), a soluble N-terminal fragment of the cluster of differentiation marker protein 14 (CD14), has been proposed as an alternative biomarker to PCT because of its higher accuracy in the identification and prognostic prediction of sepsis/septic shock [55,56]. However, because of higher costs and lower laboratory availability, PSP remains less tested than PCT.

Since any antimicrobial administration should be based on local epidemiology, we propose a model that provides a summary of the main antibiotic therapies according to the infection site (Table 2) [57,58,59,60,61,62,63,64,65,66]. As described above, for patients with septic shock, it might be advisable to start with multi-antimicrobial regimens with a wide spectrum of activity (as indicated in the last two columns of Table 2). Moreover, the use of echinocandins (e.g., caspofungin) may be considered in suspected febrile invasive candidiasis or other potentially life-threatening mycoses, particularly with respect to immunocompromised patients [67]. Considering the duration of empirical antimicrobial treatments, various randomized clinical trials (RCTs) have shown no differences in mortality between short- vs. long-term therapies [68,69,70,71,72,73,74], which has provoked the surviving sepsis campaign (SSC) to recommend shorter treatments [2]. Furthermore, there is direct evidence that PCT should guide treatment duration [75,76,77,78,79,80,81,82,83,84,85,86,87,88].

#### Emergency Physician’s Point of View

Appropriate cultural samples are required before antibiotic therapy is started. This treatment should be based on clinical/epidemiological criteria and be administrated promptly, ideally within 1 h. A frequent re-assessment of patients’ condition and PCT levels is advisable to plan an adequate reduction strategy. When possible, short courses of antimicrobial treatments may be indicated.

### 3.2. Fluids

The second pillar of treatment is fluid resuscitation. Sepsis is accompanied by severe vasoplegia, which is secondary to the shedding of the glycocalyx, an affliction that may lead to distributive shock. The effective support of hemodynamic functions is essential for the survival of patients with sepsis/septic shock [89]. In the past, the “ideal” treatment for a septic patient was based on massive volume replenishment [90,91]. Recently, this approach has been questioned. Indeed, due to hemodynamic uncoupling, microcirculation perfusion does not necessarily improve with the stabilization of cardiovascular parameters; moreover, glycocalyx abnormalities and endothelial dysfunction can even be worsened by aggressive treatments [92,93,94,95].

#### 3.2.1. Type of Fluids

The two main types of resuscitation fluids are isotonic crystalloids and colloids. The following paragraphs will describe the main features of these therapies.

##### Crystalloids

Crystalloids are divided into two main categories (i.e., chloride-rich solutions and balanced crystalloids); according to the previous guidelines, they should be considered the fluids of choice in patients with sepsis/septic shock [2,96]. The administration of balanced crystalloids for the fluid resuscitation of septic patients is preferable for two reasons: (i) they have an electrolytic composition closer to that of plasma, and (ii) chloride-rich solutions are associated with a high risk of hyperchloremic acidosis (especially in large volumes). To date, the volume of fluids to be infused in a septic patient in the early stages of treatment is largely debated and, therefore, remains incompletely defined [97]. Further discussion about the amount of fluid will be discussed in a separate section given below.

##### Colloids

In the past, the fluids of choice were colloids (e.g., hydroxyethyl-starch (HES), gelatines, and dextrans), as higher-weight molecules were thought to reduce extravascular leakage and increase long-term intravascular volume [7,98,99]. However, since the integrity of the glycocalyx is altered in septic patients, the actual intravascular volume of these fluids is apparently less than expected [99,100,101]. Moreover, no data have consistently demonstrated the superiority of colloids over crystalloids with respect to reducing mortality for sepsis [7]. Different studies have highlighted an increased risk of tubular necrosis and acute kidney injury (AKI) after treatment with colloids [102,103,104]. Therefore, the safety committee of the European Medicines Agency (EMA) has recommended that authorization of the marketing of HES solutions should be suspended in Europe.

Albumin

The use of albumin in sepsis treatment has been largely debated [105]. Despite the theoretical advantage of albumin over crystalloids in maintaining oncotic pressure, multiple RCTs and meta-analyses have reported that albumin infusion did not improve either short- or long-term mortality [106,107,108,109,110].

#### 3.2.2. Amount of Fluids

The total amount of fluid that should be administered in septic patients for proper resuscitation is still debated. The SSC suggests (but previously strongly recommended) treating septic subjects with at least 30 mL/kg of intravenous (IV) crystalloids within the first 3 h [2]. This volume has been strongly debated in the last years [111,112,113,114,115], for which the common conclusion was to perform an individualized treatment targeted toward “glycocalyx resuscitation” according to fluid tolerance (FT) and fluid responsiveness (FR) [116,117]. FT can be expressed as the degree to which a patient can tolerate the administration of fluids without the onset of organ dysfunction [118]. FR is commonly defined as a stroke volume (SV) increase of at least 10% following a fluid bolus of 200–500 mL in 10–15 min [119,120,121]. Furthermore, there are different articles of evidence in the literature showing that fluid overload can damage the glycocalyx, leading to poor clinical outcomes [101,122,123,124]. In the last few years, different methods have been proposed to establish and monitor FR (e.g., passive leg raise SPLR), SV, and the collapsibility index of inferior vena cava (CI-IVC)), but a consensus has not yet been reached [125]. However, the general agreement among experts favors the use of dynamic tools instead of static ones [117,119,125]. Similarly, the main resuscitation endpoints are progressively evolving toward restoring microcirculation [117]. In 2018, Perner et al. proposed an individualized fluid treatment based on a repeated bolus of 250–500 mL of IV crystalloids with the continuous monitoring of FR and the early administration of vasopressors if circulation fails to improve [126]. However, a recent RCT demonstrated that the restrictive vs. liberal fluid strategies did not significantly differ in terms of 90-day mortality among patients with sepsis-induced hypotension [127].

##### Emergency Physician’s Point of View

Balanced crystalloids are the fluid of choice. Since it is impractical to standardize the amount of fluid according to each patient, an individualized strategy of resuscitation based on FT and FR is preferable. Since the clinical evidence is equivocal and no differences have been shown with respect to restrictive vs. liberal fluid strategies, we consider it to be reasonable to adopt an approach based on small and repeated boluses (250–500 mL) of crystalloids with continuous hemodynamic monitoring to avoid fluid overload.

### 3.3. Vasoactive Agents

The use of inotropic drugs represents one of the cornerstones of septic shock treatment. The pathogenesis of this severe and life-threatening condition is closely related to the loss of vasomotor tone with consequent systemic vasodilation and hypotension [89,128]. Since an MAP of 60 to 65 mmHg is considered a threshold for an increased risk of morbidity and mortality, the SSC recommends an MAP target of 65 mmHg and indicates norepinephrine (NE) as the first-choice drug [2]. Recent RCTs have proposed a “permissive hypotension” (MAP 60–65 mmHg) in patients ≥65 years with septic shock showing no differences in 90-day mortality, whereas higher blood pressure values (≥65 mmHg) do not seem to add further benefits [129,130].

NE is an α-1/β-1 adrenergic agonist that predominantly manifests its effects at the vascular level, enhancing vascular filling pressure and redistributing blood flow via its venoconstrictive effect [131]. In addition, it improves myocardial contractility and cardiac output (increasing preload) while having a minor impact on heart rate [132]. Ideally, an inotropic drug assessment should occur within the first hour if fluid infusion alone is not sufficient to reach the desired MAP [2]. Various studies have demonstrated that early NE administration (at a dose of 0.1–1.2 μg/kg/min) may improve the outcomes of septic patients, although the results remain controversial. In particular, it has been shown to be effective in shortening length of stay (LOS) and reducing mortality [133,134,135,136,137,138,139,140]. Since the β-adrenergic component of cardiomyocytes has not yet been altered in the early stages of shock, prompt NE infusion improves coronary perfusion by increasing atrial diastolic pressure [141]. In addition, early inotropic administration seems to successfully resuscitate microcirculation, with a consequent improvement in tissue perfusion and oxygenation [142]. Finally, through its vasoactive effects on peripheral circulation, NE allows for the administration of a smaller crystalloid amount, thus circumventing the risk of fluid overload [142,143].

Vasopressin (VP) may be considered a second-line choice for septic shock treatment [2]. According to the SSC’s recommendations, it can be administered (at a dose of 0.25–0.5 μg/kg/min) in addition to NE to obtain the target MAP by decreasing the dosage of the latter and reducing the side effects due to adrenergic overload [2]. Furthermore, two randomized studies have shown that its efficacy (when used alone) is greater than that of NE in less-severe cases of septic shock, thereby facilitating the earlier attainment of the pressure target. The goal is not only to resuscitate the cardiovascular system but also to limit the side effects due to adrenergic overload [132,144]. However, two recent meta-analyses assessing the effect of VP administration concluded that its early initiation was not associated with a decrease in short-term mortality, a shorter ICU length of stay, or LOS, but can reduce the use of renal replacement therapy (RRT) [145,146].

Epinephrine should be considered as a third-line treatment for septic shock, and its use should be limited to those cases with inadequate MAP levels despite NE and VP administration [2]. As for VP, it can be used concomitantly with NE. Due to its important β-adrenergic effect, the use of epinephrine is indicated to a greater extent in cases of cardiac dysfunction [147]. Furthermore, its administration may lead to more side effects than those induced by NE (e.g., tachycardia, tachyarrhythmia, and increased blood lactate concentrations [132,148].

Many authors have proposed early vasopressor administration in patients with septic shock [108,132,140,149,150,151,152,153], even in pre-hospital settings [139]. The results are still debated, although it seems that early NE treatment might reduce fluid overload and improve patients’ outcomes.

#### Emergency Physician’s Point of View

Vasopressors should be administered in cases of an MAP < 65 mmHg despite fluid replacement. NE (at a dose of 0.1–1.2 μg/kg/min) is the drug of choice for septic patients, and its early administration could prevent fluid overload, thus reducing mortality. VP (at a dose of 0.25–0.5 μg/kg/min) might be associated with NE when target MAP is not achieved.

### 3.4. Oxygenation and Ventilation Support

#### 3.4.1. Oxygen

Oxygen represents the most common treatment administered to any patient with a medical emergency, including those with sepsis/septic shock [2,154]. In clinical practice, oxygen is overused, often leading to hypoxemia, which may negatively impact patients’ survival. While several studies have demonstrated a correlation between hypoxemia and increased mortality in patients who have suffered from a stroke, traumatic brain injury, or cardiac arrest, this relation is not clear in subjects with sepsis/septic shock [155]. The latest SCC guidelines do not provide any recommendations for the preferential use of oxygen therapy or targets (generally defined as PaO_2_ 55 to 70 mmHg; SpO_2_ 88 to 92%) for adults [2]. A recent meta-analysis concluded that there is low/very low evidence regarding an optimal oxygenation strategy for acutely ill adults. However, only two out of the fifty analyzed trials included patients with sepsis/septic shock [156].

#### 3.4.2. Ventilation

Since the publication of SSC guidelines, no new data regarding the benefit of non-invasive ventilation (NIV) over mechanical ventilation (MV) have been collected or reviewed; thus, no updated recommendations can be provided. Two recent systematic reviews explored the use of low-tidal-volume ventilation (LTVV), proposing a reduction in tidal volume from 10 to 6 mL/kg for septic patients at the ED [157,158]. Both studies concluded that the use of LTVV is associated with improved clinical outcomes for mechanically ventilated ED patients. However, an individualized and cautious ventilatory approach should be considered for patients with severe metabolic acidosis [2]. Moreover, the application of elevated intrathoracic pressure from NIV/MV can have a significant effect on cardiovascular function, reducing venous return and, consequently, cardiac output [159]. Therefore, to avoid this effect, the use of a high-flow nasal cannula (HFNC) has been proposed for patients with sepsis and acute hypoxic respiratory failure.

#### 3.4.3. High-Flow Nasal Cannula

An HFNC provides heated and humidified oxygen at high flow rates, generating low levels of positive pressure in the upper airways. Treatment with an HFNC induces multiple effects, including increased oxygenation, lower respiratory rates, and reduced inspiratory effort, thus improving survival rates for patients with acute hypoxic respiratory failure [160,161].

Despite the increasing use of HFNCs for critically ill patients, there are no consistent data on their efficacy with respect to sepsis/septic shock as their use was quite limited when the SCC guidelines were issued. Despite the low quality of the evidence, the SSC suggested HFNC application rather than NIV in septic patients with acute hypoxic respiratory failure [2]. Recent data support the use of an HFNC in this subset, especially during weaning phases from mechanical ventilation or when preventing reintubation [162,163]. However, Kim et al. emphasized the need for close patient monitoring since an HNFC may fail to prevent intubation or increase survival rates [164].

##### Emergency Physician’s Point of View

Oxygen therapy should be started at 15 L/min via a reservoir mask and titrated to aim toward SpO_2_ 94–98% or SpO_2_ 88–92% if the patient is at risk of hypercapnic respiratory failure (e.g., they have a history of chronic obstructive pulmonary disease, severe obesity, etc.). For patients on NIV/MV, we suggest a low tidal volume (6 mL/kg). An HFNC may be successfully used in septic patients with hypoxic respiratory failure.

### 3.5. Other Treatments

#### 3.5.1. Heparin

Since critically ill patients are at high risk for deep vein thrombosis and pulmonary embolism, heparin should be included in the treatment of these cases. Furthermore, sepsis/septic shock might induce disseminated intravascular coagulation, a life-threatening complication characterized by the suppression of fibrinolysis, which often leads to multiple organ failure [165]. The SSC guidelines strongly recommended venous thromboembolism (VTE) prophylaxis via administrating low-molecular-weight heparin (LMWH) instead of unfractionated heparin (UFH) [2]. Furthermore, various studies have proven that heparin can induce other significant effects (i.e., anti-inflammatory effects, anti-complemental activation, and the modulation of various proteases) rather than solely prophylaxis (via anti-coagulation) in septic patients sepsis [166,167]. Moreover, there is increasing evidence suggesting that heparin might mitigate pulmonary hypertension by interrupting neutrophil adhesion to the lung endothelium, thus reducing neutrophil migration into the interstitial space (which ultimately leads to decreased edema) [168,169,170]. Mechanical VTE prophylaxis should be considered in patients with sepsis/septic shock for whom pharmacologic prophylaxis is contraindicated [171]. To date, no evidence on the use of direct oral anticoagulant treatment in VTE prophylaxis has been produced.

##### Emergency Physician’s Point of View

VTE prophylaxis should be administered to sepsis/septic shock patients, preferably using LMWH (rather than UFH); mechanical prophylaxis may be advised for the treatment of patients with absolute contraindications to heparin treatment.

#### 3.5.2. Insulin

Stress hyperglycemia, due to increased glucocorticoid and catecholamine release and insulin resistance, is a common effect and may worsen septic patients’ outcome [172,173,174,175]. In critically ill patients, insulin infusion should always be selected over oral anti-diabetic treatments [176]. Rim et al. evaluated the risk of sepsis among patients treated with different oral hypoglycemic therapies and showed that metformin, compared to meglitinides and various inhibitors (sodium-glucose cotransporter-2, alpha-glucosidase inhibitors, and dipeptidyl-peptidase 4), was associated with a lower risk of hospital admission for infection [177]. Since septic patients often show frequent variations in glycemic values, the use of a careful monitoring strategy is advisable [178].

##### Emergency Physician’s Point of View

According to the SSC guidelines, glycemic control (with a glucose target between 144 to 180 mg/dL), preferably via insulin administration, is highly recommended for septic patients [2].

#### 3.5.3. Proton Pump Inhibitors

In its 2016 guidelines, the SSC strongly recommended the use of stress ulcer prophylaxis for septic patients [1]. This recommendation was downgraded in 2021 because of weak evidence regarding the benefit–risk ratio [2]. Various studies have demonstrated that proton pump inhibitors (PPIs) do not significantly improve critical patients’ prognosis, leading to a modest reduction in gastrointestinal (GI) bleeding [179,180]. Furthermore, Huang et al. proved that among adult septic patients at risk for GI bleeding or stress ulcers, PPI treatment, with more than histamine-2 receptor blockers, increased rates of in-hospital mortality, bleeding, and pneumonia [181]. A recent meta-analysis reported that PPIs in hospitalized patients were associated with recurrent *Clostridioides difficile* infections [182]. Although adverse effects have been reported in critically ill patients, the evidence that has accumulated thus far is not strong enough to discourage the use of PPIs in sepsis treatment [2]. In addition, stress ulcer prophylaxis is inexpensive, requires limited resources, and is abundantly applicable (even in low-income countries) [2,179].

##### Emergency Physician’s Point of View

The current evidence does not provide any further information about PPI assessment for stress ulcer prophylaxis in patients with sepsis/septic shock. Therefore, in line with the SSC’s guidelines, PPI treatment should be pursued.

#### 3.5.4. Renal Replacement Therapy

Acute kidney injury (AKI) is defined as an increase in serum creatinine by ≥0.3 mg/dL within 48 h or by ≥1.5 mg/dL from the baseline values within the previous 7 days or a decrease in urine volume < 0.5 mL/kg/h after 6 h, and it should be stratified for severity according to serum creatinine or urine output [183]. AKI is a common complication affecting about 40% and up to 64% of septic and septic shock patients, respectively, thus increasing mortality rates [184,185,186]. RRT is commonly required in septic AKI associated with other absolute indications for dialysis (e.g., severe metabolic acidosis, refractory fluid overload, electrolyte imbalance, and uremic complications) [2,187]. RRT techniques include continuous RRT and intermittent hemodialysis (IHD); however, which one is the best modality for optimal RRT in septic AKI remains unsettled [188]. Since high-quality RCTs and meta-analyses have reported contradictory results, the timing of RRT initiation is still controversial [188,189]. So far, there has only been one RCT incorporating septic patients with AKI, which concluded that there is no significant difference in overall mortality at 90 days between patients who had undergone early vs. delayed RRT [190]. The CRTSAKI Study (Continuous RRT Timing in Sepsis-associated AKI in ICU), comparing early vs. delayed RRT strategies with respect to the outcomes of patients with septic AKI in the ICU, is in progress, and the results are eagerly awaited [191].

##### Emergency Physician’s Point of View

Even though AKI is a common complication in septic patients, sepsis alone is not an indication for RRT. Thus, we suggest referring to specific AKI guidelines for this highly debated issue [183].

#### 3.5.5. Steroids

Since a pro-inflammatory state and the cytokine cascade are thought to contribute significantly to the manifestation of sepsis, various studies have proposed the use of steroid treatments; however, the data supporting the use of these drugs remain inconclusive [192,193,194]. So far, only hydrocortisone (at a dose of 200 mg/die) has been suggested by the SSC for adult septic shock patients not reaching the target MAP despite vasopressor administration [2,195,196]. In a recent meta-analysis involving over 9000 subjects, Fong et al. showed that a glucocorticoid shortened the time to the resolution of septic shock and the duration of MV while not affecting LOS or mortality. Notably, the combination of a glucocorticoid and fludrocortisone improved short- and longer-term mortality [197].

##### Emergency Physician’s Point of View

Despite the role of steroids emphasized by Fong et al., the routine table use of glucocorticoids (alone or in combination with fludrocortisone) in septic shock management is not adequately supported by the current evidence. The use of hydrocortisone may be considered for patients with a vasopressor-resistant, inadequate MAP.

#### 3.5.6. Sodium Bicarbonate

Sepsis and septic shock may induce acidosis through different pathophysiological mechanisms, which mainly lead to lactic or metabolic acidosis [198,199,200]. The role of sodium bicarbonate in these conditions has been largely debated, but no clear results have been obtained. In particular, the role of bicarbonate therapy in patients with lactic acidosis is controversial. Most experts believe that this treatment is appropriate in cases of severe lactic acidosis with acidemia (arterial pH < 7.1) which may lead to hemodynamic instability as a result of reduced left ventricular contractility, arterial vasodilation, and impaired responsiveness to catecholamines [201].

The clinical impacts and treatment of severe acute metabolic acidosis caused by sepsis/septic shock remain controversial, and experts disagree about the indications for the use of sodium bicarbonate [202].

A recent study by Zhang et al. involving a total of 1718 septic patients with metabolic acidosis subdivided into two subgroups (i.e., 500 subjects treated with sodium bicarbonate vs. 1218 untreated) showed that the treated patients did not present decreased mortality. However, an improvement in the survival of septic patients with AKI stage 2 or 3 and severe acidosis was observed [203]. Based on the most recent studies, most physicians agree that the treatment of metabolic acidosis should be initiated when bicarbonate levels are <5 mEq/L and pH is <7.1 [204]. Bicarbonate therapy in patients with less severe acidosis (pH 7.1 or greater) is not recommended unless the patient also has severe acute kidney injury [202,205]. In this regard, a multicenter, open-label, randomized controlled phase 3 trial proposed that early sodium bicarbonate infusion would result in lower 28-day mortality from any cause and lower organ failure incidence at 7 days after ICU admission for patients with severe metabolic acidemia. The study was performed by screening 26 ICUs and enrolling 389 patients in an intention-to-treat analysis (194 in the control group and 195 in the bicarbonate group). The authors concluded that sodium bicarbonate had no effect on reducing 28-day mortality or 7-day risk of organ failure; nonetheless, the treatment seemed to decrease the need for RRT and the a priori defined mortality of patients with AKI [206].

##### Emergency Physician’s Point of View

Despite controversial evidence, sodium bicarbonate is a reasonable treatment for septic patients with severe metabolic/lactic acidosis (bicarbonate levels <5 mEq/L and/or pH < 7.1) or an AKI stage 2 or 3. Therefore, this therapy should be indicated as a bridge to be crossed before the main pillars of treatment begin to be effective.

#### 3.5.7. Acetaminophen

This drug effectively reduces temperature in non-neurocritical ill patients but does not change mortality or other outcomes; therefore, it should not be considered one of the main pillars of sepsis treatment [207,208].

##### Emergency Physician’s Point of View

Acetaminophen is not considered a pillar of sepsis treatment and should be administered as a symptomatic drug.

## 4. Conclusions

Sepsis is a life-threatening and time-dependent condition that is still accompanied by an overall poor prognosis. Several reasons may be advocated to explain why sepsis and septic shock challenge emergency physicians in daily practice, including (i) its insidious clinical onset; (ii) misdiagnosis leading to delayed treatment and subsequent worsening of clinical outcomes and quality of life; and finally (iii) multidisciplinary and challenging management with different therapeutic aspects that are still debated, e.g., the time until antimicrobic treatment, adequate fluid resuscitation, early vasopressor administration, and oxygen targets. Nonetheless, a well-orchestrated treatment based on selected antimicrobics, fluids, oxygen, and, if necessary, vasoactive agents can improve patients’ outcomes. Taken together, the data presented in this review on sepsis management (summarized in Table 3) provide a strong basis for minimizing the current unmet needs of this severe condition.

## Figures and Tables

**Table 1:** Main risk factors for multi-drug resistant pathogens.

***MRSA***	Previous infection/colonization by MRSA in the last 12 months Hemodialysis or peritoneal dialysis Presence of central venous catheters or intravascular devices Administration of multiple antibiotics in the last 30 days (in particular with cephalosporins or fluoroquinolones) Immunodepression Immunosuppressor treatments Rheumatoid arthritis Drug addiction Patients coming from long-term care facilities or who have undergone hospital stay in the last 12 months Close contact with patients colonized by MRSA
***ESBL***	Previous infection/colonization with ESBL in the last 12 months Prolonged hospitalization (>10 days, in particular in ICU/hospice/long-term care facilities) Presence of permanent urinary catheter Administration of multiple antibiotics in the last 30 days (particularly with cephalosporins or fluoroquinolones) Patients with percutaneous endoscopic gastrostomy
***Pseudomonas aeruginosa***	Previous infection/colonization with P. aeruginosa in the last 12 months Administration of multiple antibiotics in the last 30 days (particularly with cephalosporins or fluoroquinolones) Pulmonary anatomic abnormalities with recurrent infections (e.g., bronchiectasis) Elderly patients (>80 years) Scarce glycemic control in diabetic subjects Presence of permanent urinary catheter Prolonged steroid use (>6 weeks) Neutropenic fever Cystic fibrosis
***Candida* spp.**	Immunodepression Presence of central venous catheters or intravascular devices Patients in total parenteral nutrition Prolonged hospitalization (>10 days, particularly in an ICU) Recent surgery (particularly abdominal surgery) Prolonged wide-range antibiotic administration Previous necrotizing pancreatitis Recent fungal infection/colonization

Note: ESBL: Extended Spectrum Beta-lactamase; ICU: Intensive Care Unit; MRSA: Methicillin-Resistant *Staphylococcus aureus*.

**Table 2:** Main empiric antimicrobic therapies according to the site of infection.

Infection Site		I Choice	II Choice	Allergy to Penicillin	Risk Factors for ESBL+	Risk Factors for MRSA
**Pulmonary** **[57,58]**	**CAP**	Amoxicillin/Clavulanate 2.2 g/tid + Azithromycin 500 mg/die or Clarithromycin 500 mg/bid	Levofloxacin 750 mg/die	Levofloxacin 750 mg/die	Piperacillin/Tazobactam 9 g LD followed by 18 g/die + Levofloxacin 750 mg/die or Meropenem 2 g LD followed by 2 g/tid	Levofloxacin 750 mg/die + Linezolid 600 mg/bid or Vancomycin 25–30 mg/kg LD than 20 mg/kg/bid
	**HAP**	Piperacillin/Tazobactam 9 g LD followed by 18 g/die or Cefepime 1 g LD followed by 2 g/tid + Linezolid 600 mg/bid	Levofloxacin 750 mg/die + Linezolid 600 mg/bid	Levofloxacin 750 mg/die + Linezolid 600 mg/bid	Piperacillin/Tazobactam 9 g LD followed by 18 g/die + Meropenem 2 g LD followed by 2 g/tid	Piperacillin/Tazobactam 9 g LD followed by 18 g/die or Cefepime 1 g LD followed by 2 g/tid + Gentamicin 5–7 mg/kg/die + Linezolid 600 mg/bid or Vancomycin 25–30 mg/kg LD than 20 mg/kg/bid
	**VAP**	Piperacillin/Tazobactam 9 g LD followed by 18 g/die or Cefepime 1 g LD followed by 2 g/tid + Linezolid 600 mg/bid	Levofloxacin 750 mg/die + Linezolid 600 mg/bid	Levofloxacin 750 mg/die + Linezolid 600 mg/bid	Piperacillin/Tazobactam 9 g LD followed by 18 g/die + Meropenem 2 g LD followed by 2 g/tid	Piperacillin/Tazobactam 9 g LD followed by 18 g/die or Cefepime 1 g LD followed by 2 g/tid + Linezolid 600 mg/bid or Vancomycin 25–30 mg/kg LD than 20 mg/kg/bid
**Urinary** **[59]**	**Community**	Piperacillin/Tazobactam 9 g LD followed by 18 g/die	Ciprofloxacin 500 mg/bid	Ciprofloxacin 500 mg/bid	Piperacillin/Tazobactam 9 g LD followed by 18 g/die	Piperacillin/Tazobactam 9 g LD followed by 18 g/die or Meropenem 2 g LD followed by 2 g/tid
	**Nosocomial**	Piperacillin/Tazobactam 9 g LD followed by 18 g/die	Meropenem 2 g LD followed by 2 g/tid	Meropenem 2 g LD followed by 2 g/tid	Meropenem 2 g LD followed by 2 g/tid	Meropenem 2 g LD followed by 2 g/tid
**Abdominal** **[60,61]**	**Community**	Amoxicillin/Clavulanate 2.2 g/tid or Ceftriaxone 2 g/die + Metronidazole 500 mg/qid	Piperacillin/Tazobactam 9 g LD followed by 18 g/die	Ciprofloxacin 500 mg/bid + Metronidazole 500 mg/qid	Meropenem 2 g LD followed by 2 g/tid	Meropenem 2 g LD followed by 2 g/tid + Vancomycin 25–30 mg/kg LD than 20 mg/kg/bid
	**Nosocomial**	Piperacillin/Tazobactam 9 g LD followed by 18 g/die	Meropenem 2 g LD followed by 2 g/tid	Ciprofloxacin 500 mg/bid + Metronidazole 500 mg/qid	Meropenem 2 g LD followed by 1 g/tid	Meropenem 2 g LD followed by 2 g/tid + Tigecycline 100 mg LD followed by 100 mg/bid ± Caspofungin 70 mg LD followed by 50 mg/die
**CNS** **[62]**	**<50 years**	Dexamethasone 0.1 mg/kg/qid + Ceftriaxone 2 g/die ± Acyclovir 10 mg/kg/tid	Dexamethasone 0.1 mg/kg/qid + Meropenem 2 g LD followed by 2 g/tid ± Acyclovir 10 mg/kg/tid	Dexamethasone 0.1 mg/kg/qid + Meropenem 2 g LD followed by 2 g/tid ± Acyclovir 10 mg/kg/tid	/	/
	**>50 years**	Dexamethasone 0.1 mg/kg/qid + Ceftriaxone 2 g/die + Ampicillin 12 g/die ± Acyclovir 10 mg/kg/tid	Dexamethasone 0.1 mg/kg/qid + Meropenem 2 g LD followed by 2 g/tid ± Acyclovir 10 mg/kg/tid	Dexamethasone 0.1 mg/kg/qid + Meropenem 2 g LD followed by 2 g/tid ± Acyclovir 10 mg/kg/tid	/	/
**Skin** **[63,64]**	**Cellulitis**	Amoxicillin/Clavulanate 2.2 g/tid ± Clindamycin 600 mg/qid	Ceftriaxone 2 g/die	Levofloxacin 750 mg/die	Piperacillin/Tazobactam 9 g LD followed by 18 g/die + Meropenem 2 g LD followed by 2 g/tid	Daptomycin 8–10 mg/kg/die or Vancomycin 25–30 mg/kg LD than 20 mg/kg/bid
	**NF**	Daptomycin 8–10 mg/kg/die + Clindamycin 600 mg/qid + Piperacillin/Tazobactam 9 g LD followed by 18 g/die	/	Daptomycin 8–10 mg/kg/die + Clindamycin 600 mg/qid + Meropenem 2 g LD followed by 2 g/tid	Daptomycin 8–10 mg/kg/die + Clindamycin 600 mg/qid + Meropenem 2 g LD followed by 2 g/tid	Daptomycin 8–10 mg/kg/die + Clindamycin 600 mg/qid + Meropenem 2 g LD followed by 2 g/tid
**Gyn** **[65]**		Clindamycin 600 mg/qid + Gentamicin 5–7 mg/kg/die	/	Clindamycin 600 mg/qid + Gentamicin 5–7 mg/kg/die	Meropenem 2 g LD followed by 2 g/tid	Meropenem 2 g LD followed by 2 g/tid
**Undefined** **[66]**		Piperacillin/Tazobactam 9 g LD followed by 18 g/die + Daptomycin 8–10 mg/kg/die or Vancomycin 25–30 mg/kg LD than 20 mg/kg/bid ± Caspofungin 70 mg LD followed by 50 mg/die	Daptomycin 8–10 mg/kg/die or Vancomycin 25–30 mg/kg LD than 20 mg/kg/bid + Meropenem 2 g LD followed by 2 g/tid ± Caspofungin 70 mg LD followed by 50 mg/die	Daptomycin 8–10 mg/kg/die or Vancomycin 25–30 mg/kg LD than 20 mg/kg/bid + Meropenem 2 g LD followed by 2 g/tid ± Caspofungin 70 mg LD followed by 50 mg/die	Daptomycin 8–10 mg/kg/die or Vancomycin 25–30 mg/kg LD than 20 mg/kg/bid + Meropenem 2 g LD followed by 2 g/tid ± Caspofungin 70 mg LD followed by 50 mg/die	Daptomycin 8–10 mg/kg/die or Vancomycin 25–30 mg/kg LD than 20 mg/kg/bid + Meropenem 2 g LD followed by 2 g/tid ± Caspofungin 70 mg LD followed by 50 mg/die

Note: Bid: bis in die; CAP: community-acquired pneumonia; CNS: central nervous system; HAP: hospital-acquired pneumonia; LD: loading dose; NF: necrotizing fasciitis; qid: quarter in die; tid: tris in die; VAP: ventilator-associated pneumonia.

**Table 3:** Summary of Emergency Physician’s perspectives reported in this manuscript.

Pillars of Treatment	Emergency Physician’s Perspectives
**Antimicrobials**	- Culture samples are required before administration of antimicrobials; - Treatments should be based on clinical/epidemiological criteria and promptly started; - Frequent re-assessments of patients’ condition and PCT levels are advisable for an adequate reduction strategy; - Short courses of antimicrobial treatments may be indicated.
**Fluids**	- Balanced crystalloids are the fluid of choice; - Individualized resuscitation strategies based on FT and FR are preferable; - Approaches based on small and repeated boluses (250–500 mL) of crystalloids with continuous hemodynamic monitoring are advised.
**Vasoactive Agents**	- Vasopressors are required if a patient’s MAP is <65 mmHg despite fluid replacement; - NE at a dose of 0.1–1.2 μg/kg/min is the drug of choice for septic patients; - Early administration of NE could prevent fluid overload, thereby reducing mortality; - VP at a dose of 0.25–0.5 μg/kg/min may be combined with NE if target MAP is not achieved.
**Oxygenation and Ventilation Support**	- Oxygenation should be started at 15 L/min via a reservoir mask; - The target values for titration should be SpO_2_ 94–98% or SpO_2_ 88–92% if the patient is at risk of hypercapnic respiratory failure; - If NIV/MV is needed, a low tidal volume (6 mL/kg) is advisable; - HFNC may be used in septic patients with hypoxic respiratory failure.
**Other Treatments**	(1) Heparin - LMWH rather than UFH should be used to prevent VTE; - Mechanical prophylaxis is advised for patients unsuitable for heparin treatment. (2) Insulin - The use of insulin is advisable to achieve a glucose target between 144–180 mg/dL. (3) Proton Pump Inhibitors - PPI treatment may be necessary to prevent stress ulcers. (4) Renal Replacement Therapy - Although AKI is a common complication of sepsis, RRT may only be indicated in some subsets of patients. (5) Steroids - Hydrocortisone may be considered in patients with vasopressor-resistant, inadequate MAP. (6) Sodium Bicarbonate - Sodium bicarbonate may be given to patients with severe bicarbonate levels < 5 mEq/L and/or pH < 7.1 or AKI stage 2 or 3. (7) Acetaminophen - Acetaminophen should be administered as a symptomatic drug.

Note: AKI: acute kidney injury; FR: fluid responsiveness; FT: fluid tolerance; HFNC: high-flow nasal cannula; LMWH: low-molecular-weight heparin; MAP: mean arterial pressure; NE: norepinephrine; PCT: procalcitonin; PPI: proton pump inhibitor; RRT: renal replacement therapy; SSC: surviving sepsis campaign; UFH: unfractionated heparin; VP: vasopressin; VTE: venous thromboembolism.

## Data Availability

There are no data available for this paper.

## Abbreviations

AKI: acute kidney injury; CCRT: continuous renal replacement therapy; CD-14: cluster of differentiation 14; CI-IVC: collapsibility index of inferior vena cava; DIC: disseminated intravascular coagulation; DVT: deep vein thrombosis; ED: emergency department; EMA: European medicines agency; FR: fluid-responsiveness; FT: fluid-tolerance; FUO: fever of unknown origin; GI: gastrointestinal; HFNC: high-flow nasal cannula; ICU: intensive care unit; IHD: intermittent hemodialysis; IHM: in-hospital mortality; IV: intravenous; LMWH: low-molecular-weight heparin; LOS: length of stay; MAP: mean arterial pressure; MIC: minimum inhibitory concentration; MRSA: methicillin-resistant *Staphylococcus aureus*; NE: norepinephrine; PCT: procalcitonin; PE: pulmonary embolism; PLR: passive leg raise; PPI: proton pump inhibitor; PSP: presepsin; qSOFA: quick sequential organ failure assessment; RCT: randomized clinical trial; RRT: renal replacement therapy; SOFA: sequential organ failure assessment; SSC: surviving sepsis campaign; SV: stroke volume; UFH: unfractionated heparin; VP: vasopressin; VTE: venous thromboembolism; WHO: World Health Organization.

## References

[1] Singer M., Deutschman C.S., Seymour C.W., Shankar-Hari M., Annane D., Bauer M., Bellomo R., Bernard G.R., Chiche J.-D., Coopersmith C.M. (2016). The Third International Consensus Definitions for Sepsis and Septic Shock (Sepsis-3). JAMA.

[2] Evans L., Rhodes A., Alhazzani W., Antonelli M., Coopersmith C.M., French C., Machado F.R., Mcintyre L., Ostermann M., Prescott H.C. (2021). Surviving sepsis campaign: International guidelines for management of sepsis and septic shock 2021. Intensive Care Med..

[3] Gauer R., Forbes D., Boyer N. (2020). Sepsis: Diagnosis and Management. Am. Fam. Physician.

[4] Chiu C., Legrand M. (2021). Epidemiology of sepsis and septic shock. Curr. Opin. Anaesthesiol..

[5] WHO (2020). Global Report on the Epidemiology and Burden of Sepsis: Current Evidence, Identifying Gaps and Future Directions.

[6] Vakkalanka J.P., Harland K.K., Swanson M.B., Mohr N.M. (2018). Clinical and epidemiological variability in severe sepsis: An ecological study. J. Epidemiol. Community Health.

[7] Yealy D.M., Mohr N.M., Shapiro N.I., Venkatesh A., Jones A.E., Self W.H. (2021). Early care of adults with suspected sepsis in the Emergency Department and Out-of-Hospital Environment: A Consensus-Based Task Force Report. Ann. Emerg. Med..

[8] Schlapbach L.J., Kissoon N., Alhawsawi A., Aljuaid M.H., Daniels R., Gorordo-Delsol L.A., Machado F., Malik I., Nsutebu E.F., Finfer S. (2020). World Sepsis Day: A global agenda to target a leading cause of morbidity and mortality. Am. J. Physiol.-Lung Cell. Mol. Physiol..

[9] Seymour C.W., Rea T.D., Kahn J.M., Walkey A.J., Yealy D.M., Angus D.C. (2012). Severe Sepsis in Pre-Hospital Emergency Care. Am. J. Respir. Crit. Care Med..

[10] Angus D.C., van der Poll T. (2013). Severe Sepsis and Septic Shock. N. Engl. J. Med..

[11] Piechota M., Banach M., Irzmanski R., Barylski M., Piechota-Urbanska M., Kowalski J., Pawlicki L. (2007). Plasma endothelin-1 levels in septic patients. J. Intensive Care Med..

[12] Ince C. (2005). The microcirculation is the motor of sepsis. Crit. Care.

[13] Wolinsky H. (1980). A proposal linking clearance of circulating lipoproteins to tissue metabolic activity as a basis for understanding atherogenesis. Circ. Res..

[14] Belousoviene E., Kiudulaite I., Pilvinis V., Pranskunas A. (2021). Links between Endothelial Glycocalyx Changes and Microcirculatory Parameters in Septic Patients. Life.

[15] Kumar A., Roberts D., Wood K.E., Light B., Parrillo J.E., Sharma S., Suppes R., Feinstein D., Zanotti S., Taiberg L. (2006). Duration of hypotension before initiation of effective antimicrobial therapy is the critical determinant of survival in human septic shock. Crit. Care Med..

[16] Ferrer R., Martin-Loeches I., Phillips G., Osborn T.M., Townsend S., Dellinger R.P., Artigas A., Schorr C., Levy M.M. (2014). Empiric antibiotic treatment reduces mortality in severe sepsis and septic shock from the first hour: Results from a guideline-based performance improvement program. Crit. Care Med..

[17] Dugar S., Choudhary C., Duggal A. (2020). Sepsis and septic shock: Guideline-based management. Clevel. Clin. J. Med..

[18] Mandell L.A., Wunderink R.G., Anzueto A., Bartlett J.G., Campbell G.D., Dean N.C., Dowell S.F., File T.M., Musher D.M., Niederman M.S. (2007). Infectious Diseases Society of America/American Thoracic Society consensus guidelines on the management of community-acquired pneumonia in adults. Clin. Infect. Dis..

[19] Pappas P.G., Kauffman C.A., Andes D., Clancy C.J., Marr K.A., Ostrosky-Zeichner L., Reboli A.C., Schuster M.G., Vazquez J.A., Walsh T.J. (2016). Executive Summary: Clinical Practice Guideline for the Management of Candidiasis: 2016 Update by the Infectious Diseases Society of America. Clin. Infect. Dis..

[20] Mermel L.A., Allon M., Bouza E., Craven D.E., Flynn P., O’Grady N.P., Raad I.I., Rijnders B.J.A., Sherertz R.J., Warren D.K. (2010). Clinical practice guidelines for the diagnosis and management of intravascular catheter-related infection: 2009 Update by the Infectious Diseases Society of America. Clin. Infect. Dis..

[21] Solomkin J.S., Mazuski J.E., Bradley J.S., Rodvold K.A., Goldstein E.J., Baron E.J., O’Neill P.J., Chow A.W., Dellinger E.P., Eachempati S.R. (2010). Diagnosis and management of complicated intra-abdominal infection in adults and children: Guidelines by the Surgical Infection Society and the Infectious Diseases Society of America. Clin. Infect. Dis..

[22] Stevens D.L., Bisno A.L., Chambers H.F., Dellinger E.P., Goldstein E.J., Gorbach S.L., Hirschmann J.V., Kaplan S.L., Montoya J.G., Wade J.C. (2015). Practice guidelines for the diagnosis and management of skin and soft tissue infections: 2014 update by the Infectious Diseases Society of America. Clin. Infect. Dis..

[23] Paul M., Shani V., Muchtar E., Kariv G., Robenshtok E., Leibovici L. (2010). Systematic review and meta-analysis of the efficacy of appropriate empiric antibiotic therapy for sepsis. Antimicrob. Agents Chemother..

[24] Guo Y., Gao W., Yang H., Ma C., Sui S. (2016). De-escalation of empiric antibiotics in patients with severe sepsis or septic shock: A meta-analysis. Heart Lung.

[25] Uddin T.M., Chakraborty A.J., Khusro A., Zidan B.R.M., Mitra S., Emran T.B., Dhama K., Ripon M.K.H., Gajdács M., Sahibzada M.U.K. (2021). Antibiotic resistance in microbes: History, mechanisms, therapeutic strategies and future prospects. J. Infect. Public Health.

[26] Klompas M., Calandra T., Singer M. (2018). Antibiotics for sepsis-finding the equilibrium. JAMA.

[27] Prescott H.C., Iwashyna T.J. (2019). Improving sepsis treatment by embracing diagnostic uncertainty. Ann. Am. Thorac. Soc..

[28] Baggs J., Jernigan J.A., Halpin A.L., Epstein L., Hatfield K.M., McDonald L.C. (2018). Risk of Subsequent Sepsis Within 90 Days After a Hospital Stay by Type of Antibiotic Exposure. Clin. Infect. Dis..

[29] Branch-Elliman W., O’Brien W., Strymish J., Itani K., Wyatt C., Gupta K. (2019). Association of Duration and Type of Surgical Prophylaxis with Antimicrobial-Associated Adverse Events. JAMA Surg..

[30] Hranjec T., Rosenberger L.H., Swenson B., Metzger R., Flohr T.R., Politano A.D., Riccio L.M., Popovsky K.A., Sawyer R.G. (2012). Aggressive versus conservative initiation of antimicrobial treatment in critically ill surgical patients with suspected intensive-care-unit-acquired infection: A quasi-experimental, before and after observational cohort study. Lancet Infect. Dis..

[31] Ong D.S.Y., Frencken J.F., Klein Klouwenberg P.M.C., Juffermans N., van der Poll T., Bonten M.J.M., Cremer O.L., MARS consortium (2017). Short-Course Adjunctive Gentamicin as Empirical Therapy in Patients with Severe Sepsis and Septic Shock: A Prospective Observational Cohort Study. Clin. Infect. Dis..

[32] Tamma P.D., Avdic E., Li D.X., Dzintars K., Cosgrove S.E. (2017). Association of Adverse Events with Antibiotic Use in Hospitalized Patients. JAMA Intern. Med..

[33] Teshome B.F., Vouri S.M., Hampton N., Kollef M.H., Micek S.T. (2019). Duration of Exposure to Antipseudomonal β-Lactam Antibiotics in the Critically Ill and Development of New Resistance. Pharmacotherapy.

[34] Contou D., Roux D., Jochmans S., Coudroy R., Guérot E., Grimaldi D., Ricome S., Maury E., Plantefève G., Mayaux J. (2016). Septic shock with no diagnosis at 24 hours: A pragmatic multicenter prospective cohort study. Crit. Care.

[35] Rhee C., Kadri S.S., Danner R.L., Suffredini A.F., Massaro A.F., Kitch B.T., Lee G., Klompas M. (2016). Diagnosing sepsis is subjective and highly variable: A survey of intensivists using case vignettes. Crit. Care.

[36] Liu V.X., Fielding-Singh V., Greene J.D., Baker J.M., Iwashyna T.J., Bhattacharya J., Escobar G.J. (2017). The Timing of Early Antibiotics and Hospital Mortality in Sepsis. Am. J. Respir. Crit. Care Med..

[37] Peltan I.D., Brown S.M., Bledsoe J.R., Sorensen J., Samore M.H., Allen T.L., Hough C.L. (2019). ED Door-to-Antibiotic Time and Long-term Mortality in Sepsis. Chest.

[38] Abe T., Kushimoto S., Tokuda Y., Phillips G.S., Rhodes A., Sugiyama T., Komori A., Iriyama H., Ogura H., Fujishima S. (2019). Implementation of earlier antibiotic administration in patients with severe sepsis and septic shock in Japan: A descriptive analysis of a prospective observational study. Crit. Care.

[39] Gaieski D.F., Mikkelsen M.E., Band R.A., Pines J.M., Massone R., Furia F.F., Shofer F.S., Goyal M. (2010). Impact of time to antibiotics on survival in patients with severe sepsis or septic shock in whom early goal-directed therapy was initiated in the emergency department. Crit. Care Med..

[40] Ko B.S., Choi S.H., Kang G.H., Shin T.G., Kim K., Jo Y.H., Ryoo S.M., Kim Y.J., Park Y.S., Kwon W.Y. (2020). Time to Antibiotics and the Outcome of Patients with Septic Shock: A Propensity Score Analysis. Am. J. Med..

[41] Puskarich M.A., Trzeciak S., Shapiro N.I., Arnold R.C., Horton J.M., Studnek J.R., Kline J.A., Jones A.E., Emergency Medicine Shock Research Network (EMSHOCKNET) (2011). Association between timing of antibiotic administration and mortality from septic shock in patients treated with a quantitative resuscitation protocol. Crit. Care Med..

[42] Rothrock S.G., Cassidy D.D., Barneck M., Schinkel M., Guetschow B., Myburgh C., Nguyen L., Earwood R., Nanayakkara P.W.B., Nannan Panday R.S. (2020). Outcome of Immediate Versus Early Antibiotics in Severe Sepsis and Septic Shock: A Systematic Review and Meta-analysis. Ann. Emerg. Med..

[43] Ryoo S.M., Kim W.Y., Sohn C.H., Seo D.W., Koh J.W., Oh B.J., Lim K.S. (2015). Prognostic value of timing of antibiotic administration in patients with septic shock treated with early quantitative resuscitation. Am. J. Med. Sci..

[44] Weinberger J., Rhee C., Klompas M. (2020). A Critical Analysis of the Literature on Time-to-Antibiotics in Suspected Sepsis. J. Infect. Dis..

[45] Alam N., Oskam E., Stassen P.M., Exter P.V., van de Ven P.M., Haak H.R., Holleman F., Zanten A.V., Leeuwen-Nguyen H.V., Bon V. (2018). Prehospital antibiotics in the ambulance for sepsis: A multicentre, open label, randomised trial. Lancet Respir. Med..

[46] Bloos F., Rüddel H., Thomas-Rüddel D., Schwarzkopf D., Pausch C., Harbarth S., Schreiber T., Gründling M., Marshall J., Simon P. (2017). Effect of a multifaceted educational intervention for anti-infectious measures on sepsis mortality: A cluster randomized trial. Intensive Care Med..

[47] Yan S.T., Sun L.C., Jia H.B., Gao W., Yang J.P., Zhang G.Q. (2017). Procalcitonin levels in bloodstream infections caused by different sources and species of bacteria. Am. J. Emerg. Med..

[48] Uzzan B., Cohen R., Nicolas P., Cucherat M., Perret G.Y. (2006). Procalcitonin as a diagnostic test for sepsis in critically ill adults and after surgery or trauma: A systematic review and meta-analysis. Crit. Care Med..

[49] Christ-Crain M., Jaccard-Stolz D., Bingisser R., Gencay M.M., Huber P.R., Tamm M., Müller B. (2004). Effect of procalcitonin-guided treatment on antibiotic use and outcome in lower respiratory tract infections: Cluster-randomised, single-blinded intervention trial. Lancet.

[50] Peng F., Chang W., Xie J.F., Sun Q., Qiu H.B., Yang Y. (2019). Ineffectiveness of procalcitonin-guided antibiotic therapy in severely critically ill patients: A meta-analysis. Int. J. Infect. Dis..

[51] Wacker C., Prkno A., Brunkhorst F.M., Schlattmann P. (2013). Procalcitonin as a diagnostic marker for sepsis: A systematic review and meta-analysis. Lancet Infect. Dis..

[52] Jensen J.U., Hein L., Lundgren B., Bestle M.H., Mohr T.T., Andersen M.H., Thornberg K.J., Løken J., Steensen M., Fox Z. (2011). Procalcitonin-guided interventions against infections to increase early appropriate antibiotics and improve survival in the intensive care unit: A randomized trial. Crit. Care Med..

[53] Layios N., Lambermont B., Canivet J.L., Morimont P., Preiser J.C., Garweg C., Ledoux D., Frippiat F., Piret S., Giot J.B. (2012). Procalcitonin usefulness for the initiation of antibiotic treatment in intensive care unit patients. Crit. Care Med..

[54] Najafi A., Khodadadian A., Sanatkar M., Shariat Moharari R., Etezadi F., Ahmadi A., Imani F., Khajavi M.R. (2015). The Comparison of Procalcitonin Guidance Administer Antibiotics with Empiric Antibiotic Therapy in Critically Ill Patients Admitted in Intensive Care Unit. Acta Med. Iran..

[55] Shozushima T., Takahashi G., Matsumoto N., Kojika M., Okamura Y., Endo S. (2011). Usefulness of presepsin (sCD14-ST) measurements as a marker for the diagnosis and severity of sepsis that satisfied diagnostic criteria of systemic inflammatory response syndrome. J. Infect. Chemother..

[56] Velissaris D., Zareifopoulos N., Karamouzos V., Karanikolas E., Pierrakos C., Koniari I., Karanikolas M. (2021). Presepsin as a Diagnostic and Prognostic Biomarker in Sepsis. Cureus.

[57] Di Pasquale M.F., Sotgiu G., Gramegna A., Radovanovic D., Terraneo S., Reyes L.F., Rupp J., González Del Castillo J., Blasi F., Aliberti S. (2019). Prevalence and Etiology of Community-acquired Pneumonia in Immunocompromised Patients. Clin. Infect. Dis..

[58] Kabak E., Hudcova J., Magyarics Z., Stulik L., Goggin M., Szijártó V., Nagy E., Stevens C. (2019). The utility of endotracheal aspirate bacteriology in identifying mechanically ventilated patients at risk for ventilator associated pneumonia: A single-center prospective observational study. BMC Infect. Dis..

[59] Ternes B., Wagenlehner F.M.E. (2020). Guideline-based treatment of urinary tract infections. Urologe A.

[60] Tseng W.P., Chen Y.C., Yang B.J., Chen S.Y., Lin J.J., Huang Y.H., Fu C.M., Chang S.C., Chen S.Y. (2017). Predicting Multidrug-Resistant Gram-Negative Bacterial Colonization and Associated Infection on Hospital Admission. Infect. Control Hosp. Epidemiol..

[61] Augustine M.R., Testerman T.L., Justo J.A., Bookstaver P.B., Kohn J., Albrecht H., Al-Hasan M.N. (2017). Clinical Risk Score for Prediction of Extended-Spectrum β-Lactamase-Producing Enterobacteriaceae in Bloodstream Isolates. Infect. Control Hosp. Epidemiol..

[62] Wall E.C., Chan J.M., Gil E., Heyderman R.S. (2021). Acute bacterial meningitis. Curr. Opin. Neurol..

[63] Bystritsky R.J. (2021). Cellulitis. Infect. Dis. Clin. N. Am..

[64] Peetermans M., de Prost N., Eckmann C., Norrby-Teglund A., Skrede S., De Waele J.J. (2020). Necrotizing skin and soft-tissue infections in the intensive care unit. Clin. Microbiol. Infect..

[65] Shields A., de Assis V., Halscott T. (2021). Top 10 Pearls for the Recognition, Evaluation, and Management of Maternal Sepsis. Obstet. Gynecol..

[66] Niederman M.S., Baron R.M., Bouadma L., Calandra T., Daneman N., DeWaele J., Kollef M.H., Lipman J., Nair G.B. (2021). Initial antimicrobial management of sepsis. Crit. Care.

[67] Lamoth F. (2023). Novel Therapeutic Approaches to Invasive Candidiasis: Considerations for the Clinician. Infect. Drug Resist..

[68] Eliakim-Raz N., Yahav D., Paul M., Leibovici L. (2013). Duration of antibiotic treatment for acute pyelonephritis and septic urinary tract infection—7 days or less versus longer treatment: Systematic review and meta-analysis of randomized controlled trials. J Antimicrob. Chemother..

[69] Sawyer R.G., Claridge J.A., Nathens A.B., Rotstein O.D., Duane T.M., Evans H.L., Cook C.H., O’Neill P.J., Mazuski J.E., Askari R. (2015). Trial of short-course antimicrobial therapy for intraabdominal infection. N. Engl. J. Med..

[70] Pugh R., Grant C., Cooke R.P., Dempsey G. (2015). Short-course versus prolonged-course antibiotic therapy for hospital-acquired pneumonia in critically ill adults. Cochrane Database Syst. Rev..

[71] Havey T.C., Fowler R.A., Daneman N. (2011). Duration of antibiotic therapy for bacteremia: A systematic review and meta-analysis. Crit. Care.

[72] Dimopoulos G., Matthaiou D.K., Karageorgopoulos D.E., Grammatikos A.P., Athanassa Z., Falagas M.E. (2008). Short- versus long-course antibacterial therapy for community-acquired pneumonia: A meta-analysis. Drugs.

[73] Tansarli G.S., Andreatos N., Pliakos E.E., Mylonakis E. (2019). A Systematic Review and Meta-analysis of Antibiotic Treatment Duration for Bacteremia Due to Enterobacteriaceae. Antimicrob. Agents Chemother..

[74] Montravers P., Tubach F., Lescot T., Veber B., Esposito-Farèse M., Seguin P., Paugam C., Lepape A., Meistelman C., Cousson J. (2018). Short-course antibiotic therapy for critically ill patients treated for postoperative intra-abdominal infection: The DURAPOP randomized clinical trial. Intensive Care Med..

[75] Annane D., Maxime V., Faller J.P., Mezher C., Clec’h C., Martel P., Gonzales H., Feissel M., Cohen Y., Capellier G. (2013). Procalcitonin levels to guide antibiotic therapy in adults with non-microbiologically proven apparent severe sepsis: A randomised controlled trial. BMJ Open.

[76] Bloos F., Trips E., Nierhaus A., Briegel J., Heyland D.K., Jaschinski U., Moerer O., Weyland A., Marx G., Gründling M. (2016). Effect of Sodium Selenite Administration and Procalcitonin-Guided Therapy on Mortality in Patients with Severe Sepsis or Septic Shock: A Randomized Clinical Trial. JAMA Intern. Med..

[77] Bouadma L., Luyt C.E., Tubach F., Cracco C., Alvarez A., Schwebel C., Schortgen F., Lasocki S., Veber B., Dehoux M. (2010). Use of procalcitonin to reduce patients’ exposure to antibiotics in intensive care units (PRORATA trial): A multicentre randomised controlled trial. Lancet.

[78] de Jong E., van Oers J.A., Beishuizen A., Vos P., Vermeijden W.J., Haas L.E., Loef B.G., Dormans T., van Melsen G.C., Kluiters Y.C. (2016). Efficacy and safety of procalcitonin guidance in reducing the duration of antibiotic treatment in critically ill patients: A randomised, controlled, open-label trial. Lancet Infect. Dis..

[79] Deliberato R.O., Marra A.R., Sanches P.R., Martino M.D., Ferreira C.E., Pasternak J., Paes A.T., Pinto L.M., dos Santos O.F., Edmond M.B. (2013). Clinical and economic impact of procalcitonin to shorten antimicrobial therapy in septic patients with proven bacterial infection in an intensive care setting. Diagn. Microbiol. Infect. Dis..

[80] Hochreiter M., Köhler T., Schweiger A.M., Keck F.S., Bein B., von Spiegel T., Schroeder S. (2009). Procalcitonin to guide duration of antibiotic therapy in intensive care patients: A randomized prospective controlled trial. Crit. Care.

[81] Liu B.H., Li H.F., Lei Y., Zhao S.X., Sun M.L. (2013). Clinical significance of dynamic monitoring of procalcitonin in guiding the use of antibiotics in patients with sepsis in ICU. Zhonghua Wei Zhong Bing Ji Jiu Yi Xue.

[82] Nobre V., Harbarth S., Graf J.D., Rohner P., Pugin J. (2008). Use of procalcitonin to shorten antibiotic treatment duration in septic patients: A randomized trial. Am. J. Respir. Crit. Care Med..

[83] Oliveira C.F., Botoni F.A., Oliveira C.R., Silva C.B., Pereira H.A., Serufo J.C., Nobre V. (2013). Procalcitonin versus C-reactive protein for guiding antibiotic therapy in sepsis: A randomized trial. Crit. Care Med..

[84] Qu R., Ji Y., Ling Y., Ye C.Y., Yang S.M., Liu Y.Y., Yang R.Y., Luo Y.F., Guo Z. (2012). Procalcitonin is a good tool to guide duration of antibiotic therapy in patients with severe acute pancreatitis. A randomized prospective single-center controlled trial. Saudi Med. J..

[85] Schroeder S., Hochreiter M., Koehler T., Schweiger A.M., Bein B., Keck F.S., von Spiegel T. (2009). Procalcitonin (PCT)-guided algorithm reduces length of antibiotic treatment in surgical intensive care patients with severe sepsis: Results of a prospective randomized study. Langenbecks Arch. Surg..

[86] Shehabi Y., Sterba M., Garrett P.M., Rachakonda K.S., Stephens D., Harrigan P., Walker A., Bailey M.J., Johnson B., Millis D. (2014). Procalcitonin algorithm in critically ill adults with undifferentiated infection or suspected sepsis. A randomized controlled trial. Am. J. Respir. Crit. Care Med..

[87] Stolz D., Smyrnios N., Eggimann P., Pargger H., Thakkar N., Siegemund M., Marsch S., Azzola A., Rakic J., Mueller B. (2009). Procalcitonin for reduced antibiotic exposure in ventilator-associated pneumonia: A randomised study. Eur. Respir. J..

[88] Xu X.L., Yan F.D., Yu J.Q., Chen Q.H., Lin H., Zheng R.Q. (2017). Efficacy and safety of procalcitonin guidance in reducing the duration of antibiotic treatment of sepsis patients. Zhonghua Yi Xue Za Zhi.

[89] Rhodes A., Evans L.E., Alhazzani W., Levy M.M., Antonelli M., Ferrer R., Kumar A., Sevransky J.E., Sprung C.L., Nunnally M.E. (2017). Surviving Sepsis Campaign: International Guidelines for Management of Sepsis and Septic Shock: 2016. Crit. Care Med..

[90] Edwards J.D. (1993). Management of septic shock. BMJ.

[91] Tuchschmidt J., Fried J., Astiz M., Rackow E. (1992). Elevation of cardiac output and oxygen delivery improves outcome in septic shock. Chest.

[92] Dyson A., Cone S., Singer M., Ackland G.L. (2012). Microvascular and macrovascular flow are uncoupled in early polymicrobial sepsis. Br. J. Anaesth..

[93] Woodcock T.E., Woodcock T.M. (2012). Revised Starling equation and the glycocalyx model of transvascular fluid exchange: An improved paradigm for prescribing intravenous fluid therapy. Br. J. Anaesth..

[94] Alphonsus C.S., Rodseth R.N. (2014). The endothelial glycocalyx: A review of the vascular barrier. Anaesthesia.

[95] Chappell D., Bruegger D., Potzel J., Jacob M., Brettner F., Vogeser M., Conzen P., Becker B.F., Rehm M. (2014). Hypervolemia increases release of atrial natriuretic peptide and shedding of the endothelial glycocalyx. Crit. Care.

[96] Brown R.M., Wang L., Coston T.D., Krishnan N.I., Casey J.D., Wanderer J.P., Ehrenfeld J.M., Byrne D.W., Stollings J.L., Siew E.D. (2019). Balanced Crystalloids versus Saline in Sepsis. A Secondary Analysis of the SMART Clinical Trial. Am. J. Respir. Crit. Care Med..

[97] Corl K.A., Prodromou M., Merchant R.C., Gareen I., Marks S., Banerjee D., Amass T., Abbasi A., Delcompare C., Palmisciano A. (2019). The Restrictive IV Fluid Trial in Severe Sepsis and Septic Shock (RIFTS): A Randomized Pilot Study. Crit. Care Med..

[98] Starling E.H. (1896). On the absorption of fluids from the connective tissue spaces. J. Physiol..

[99] Myburgh J.A., Mythen M.G. (2013). Resuscitation fluids. N. Engl. J. Med..

[100] Tseng C.H., Chen T.T., Wu M.Y., Chan M.C., Shih M.C., Tu Y.K. (2020). Resuscitation fluid types in sepsis, surgical, and trauma patients: A systematic review and sequential network meta-analyses. Crit. Care.

[101] Annane D., Siami S., Jaber S., Martin C., Elatrous S., Declère A.D., Preiser J.C., Outin H., Troche G., Charpentier C. (2013). Effects of fluid resuscitation with colloids vs crystalloids on mortality in critically ill patients presenting with hypovolemic shock: The CRISTAL randomized trial. JAMA.

[102] Mutter T.C., Ruth C.A., Dart A.B. (2013). Hydroxyethyl starch (HES) versus other fluid therapies: Effects on kidney function. Cochrane Database Syst. Rev..

[103] Mårtensson J., Bellomo R. (2015). Are all fluids bad for the kidney?. Curr. Opin. Crit. Care.

[104] Casey J.D., Brown R.M., Semler M.W. (2018). Resuscitation fluids. Curr. Opin. Crit. Care.

[105] Mayerhöfer T., Wiedermann C.J., Joannidis M. (2021). Use of albumin: State of the art. Med. Klin. Intensivmed. Notf..

[106] Caironi P., Tognoni G., Gattinoni L. (2014). Albumin replacement in severe sepsis or septic shock. N. Engl. J. Med..

[107] Park C.H.L., de Almeida J.P., de Oliveira G.Q., Rizk S.I., Fukushima J.T., Nakamura R.E., Mourão M.M., Galas F.R.B.G., Abdala E., Pinheiro Freire M. (2019). Lactated Ringer’s Versus 4% Albumin on Lactated Ringer’s in Early Sepsis Therapy in Cancer Patients: A Pilot Single-Center Randomized Trial. Crit. Care Med..

[108] Kakaei F.H.S., Asheghvatan A., Zarrintan S., Asvadi T., Beheshtirouy S., Mohajer A. (2017). Albumin as a resuscitative fluid in patients with severe sepsis: A randomized clinical trial. Adv. Biosci. Clin. Med..

[109] Lewis S.R., Pritchard M.W., Evans D.J., Butler A.R., Alderson P., Smith A.F., Roberts I. (2018). Colloids versus crystalloids for fluid resuscitation in critically ill people. Cochrane Database Syst. Rev..

[110] Martin G.S., Bassett P. (2019). Crystalloids vs. colloids for fluid resuscitation in the Intensive Care Unit: A systematic review and meta-analysis. J. Crit. Care.

[111] Marik P.E., Byrne L., van Haren F. (2020). Fluid resuscitation in sepsis: The great 30 mL per kg hoax. J. Thorac. Dis..

[112] Chaudhuri D., Herritt B., Lewis K., Diaz-Gomez J.L., Fox-Robichaud A., Ball I., Granton J., Rochwerg B. (2021). Dosing Fluids in Early Septic Shock. Chest.

[113] Lat I., Coopersmith C.M., De Backer D., Coopersmith C.M., Research Committee of the Surviving Sepsis Campaign (2021). The surviving sepsis campaign: Fluid resuscitation and vasopressor therapy research priorities in adult patients. Intensive Care Med. Exp..

[114] Gavelli F., Castello L.M., Avanzi G.C. (2021). Management of sepsis and septic shock in the emergency department. Intern. Emerg. Med..

[115] Ladzinski A.T., Thind G.S., Siuba M.T. (2021). Rational Fluid Resuscitation in Sepsis for the Hospitalist: A Narrative Review. Mayo Clin. Proc..

[116] Trzeciak S., Cinel I., Phillip Dellinger R., Shapiro N.I., Arnold R.C., Parrillo J.E., Hollenberg S.M., Microcirculatory Alterations in Resuscitation and Shock (MARS) Investigators (2008). Resuscitating the microcirculation in sepsis: The central role of nitric oxide, emerging concepts for novel therapies, and challenges for clinical trials. Acad. Emerg. Med..

[117] Bakker J., Kattan E., Annane D., Castro R., Cecconi M., De Backer D., Dubin A., Evans L., Gong M.N., Hamzaoui O. (2022). Current practice and evolving concepts in septic shock resuscitation. Intensive Care Med..

[118] Kattan E., Castro R., Miralles-Aguiar F., Hernández G., Rola P. (2022). The emerging concept of fluid tolerance: A position paper. J Crit. Care.

[119] Monnet X., Marik P.E., Teboul J.L. (2016). Prediction of fluid responsiveness: An update. Ann. Intensive Care.

[120] Monnet X., Teboul J.-L. (2018). My patient has received fluid. How to assess its efficacy and side effects?. Ann. Intensive Care.

[121] Bentzer P., Griesdale D.E., Boyd J., MacLean K., Sirounis D., Ayas N.T. (2016). Will this hemodynamically unstable patient respond to a bolus of intravenous fluids?. JAMA J. Am. Med. Assoc..

[122] Messmer A.S., Zingg C., Müller M., Gerber J.L., Schefold J.C., Pfortmueller C.A. (2020). Fluid Overload and Mortality in Adult Critical Care Patients-A Systematic Review and Meta-Analysis of Observational Studies. Crit. Care Med..

[123] Fang J., Wang M., Gong S., Cui N., Xu L. (2022). Increased 28-day mortality due to fluid overload prior to continuous renal replacement in sepsis associated acute kidney injury. Ther. Apher. Dial..

[124] Kattan E., Ospina-Tascón G.A., Teboul J.L., Castro R., Cecconi M., Ferri G., Bakker J., Hernández G., ANDROMEDA-SHOCK Investigators (2020). Systematic assessment of fluid responsiveness during early septic shock resuscitation: Secondary analysis of the ANDROMEDA-SHOCK trial. Crit. Care.

[125] Monnet X., Shi R., Teboul J.L. (2022). Prediction of fluid responsiveness. What’s new?. Ann. Intensive Care.

[126] Perner A., Cecconi M., Cronhjort M., Darmon M., Jakob S.M., Pettilä V., van der Horst I.C.C. (2018). Expert statement for the management of hypovolemia in sepsis. Intensive Care Med..

[127] Shapiro N.I., Douglas I.S., Brower R.G., Brown S.M., Exline M.C., Ginde A.A., Gong M.N., Grissom C.K., Hayden D., National Heart, Lung, and Blood Institute Prevention and Early Treatment of Acute Lung Injury Clinical Trials Network (2023). Early Restrictive or Liberal Fluid Management for Sepsis-Induced Hypotension. N. Engl. J. Med..

[128] Vincent J.L., De Backer D. (2013). Circulatory shock. N. Engl. J. Med..

[129] Lamontagne F., Meade M.O., Hébert P.C., Asfar P., Lauzier F., Seely A.J.E., Day A.G., Mehta S., Muscedere J., Bagshaw S.M. (2016). Higher versus lower blood pressure targets for vasopressor therapy in shock: A multicentre pilot randomized controlled trial. Intensive Care Med..

[130] Lamontagne F., Richards-Belle A., Thomas K., Harrison D.A., Sadique M.Z., Grieve R.D., Camsooksai J., Darnell R., Gordon A.C., Henry D. (2020). Effect of Reduced Exposure to Vasopressors on 90-Day Mortality in Older Critically Ill Patients with Vasodilatory Hypotension: A Randomized Clinical Trial. JAMA.

[131] Hernández G., Teboul J.L., Bakker J. (2019). Norepinephrine in septic shock. Intensive Care Med..

[132] Shi R., Hamzaoui O., De Vita N., Monnet X., Teboul J.L. (2020). Vasopressors in septic shock: Which, when, and how much?. Ann. Transl. Med..

[133] Permpikul C., Tongyoo S., Viarasilpa T., Trainarongsakul T., Chakorn T., Udompanturak S. (2019). Early Use of Norepinephrine in Septic Shock Resuscitation (CENSER). A Randomized Trial. Am J Respir Crit. Care Med..

[134] Ammar M.A., Ammar A.A., Wieruszewski P.M., Bissell B.D., TLong M., Albert L., Khanna A.K., Sacha G.L. (2022). Timing of vasoactive agents and corticosteroid initiation in septic shock. Ann. Intensive Care.

[135] Alshahrani M.S., Alatigue R. (2021). Association Between Early Administration of Norepinephrine in Septic Shock and Survival. Open Access Emerg. Med..

[136] Persichini R., Silva S., Teboul J.L., Jozwiak M., Chemla D., Richard C., Monnet X. (2012). Effects of norepinephrine on mean systemic pressure and venous return in human septic shock. Crit. Care Med..

[137] Varpula M., Tallgren M., Saukkonen K., Voipio-Pulkki L.M., Pettilä V. (2005). Hemodynamic variables related to outcome in septic shock. Intensive Care Med..

[138] Li Y., Li H., Zhang D. (2020). Timing of norepinephrine initiation in patients with septic shock: A systematic review and meta-analysis. Crit. Care.

[139] Jouffroy R., Hajjar A., Gilbert B., Tourtier J.P., Bloch-Laine E., Ecollan P., Boularan J., Bounes V., Vivien B., Gueye P.N. (2022). Prehospital norepinephrine administration reduces 30-day mortality among septic shock patients. BMC Infect. Dis..

[140] Xu F., Zhong R., Shi S., Zeng Y., Tang Z. (2022). Early initiation of norepinephrine in patients with septic shock: A propensity score-based analysis. Am. J. Emerg. Med..

[141] Hamzaoui O., Jozwiak M., Geffriaud T., Sztrymf B., Prat D., Jacobs F., Monnet X., Trouiller P., Richard C., Teboul J.L. (2018). Norepinephrine exerts an inotropic effect during the early phase of human septic shock. Br. J. Anaesth..

[142] Boyd J.H., Forbes J., Nakada T.A., Walley K.R., Russell J.A. (2011). Fluid resuscitation in septic shock: A positive fluid balance and elevated central venous pressure are associated with increased mortality. Crit. Care Med..

[143] Vincent J.L., Sakr Y., Sprung C.L., Ranieri V.M., Reinhart K., Gerlach H., Moreno R., Carlet J., Le Gall J.R., Payen D. (2006). Sepsis in European intensive care units: Results of the SOAP study. Crit. Care Med..

[144] Russell J.A., Walley K.R., Singer J., Gordon A.C., Hébert P.C., Cooper D.J., Holmes C.L., Mehta S., Granton J.T., Storms M.M. (2008). Vasopressin versus norepinephrine infusion in patients with septic shock. N. Engl. J. Med..

[145] Huang H., Wu C., Shen Q., Xu H., Fang Y., Mao W. (2021). The effect of early vasopressin use on patients with septic shock: A systematic review and meta-analysis. Am. J. Emerg. Med..

[146] Sedhai Y.R., Shrestha D.B., Budhathoki P., Memon W., Acharya R., Gaire S., Pokharel N., Maharjan S., Jasaraj R., Sodhi A. (2022). Vasopressin versus norepinephrine as the first-line vasopressor in septic shock: A systematic review and meta-analysis. J. Clin. Transl. Res..

[147] Jentzer J.C., Hollenberg S.M. (2021). Vasopressor and Inotrope Therapy in Cardiac Critical Care. J. Intensive Care Med..

[148] Belletti A., Nagy A., Sartorelli M., Mucchetti M., Putzu A., Sartini C., Morselli F., De Domenico P., Zangrillo A., Landoni G. (2020). Effect of Continuous Epinephrine Infusion on Survival in Critically Ill Patients: A Meta-Analysis of Randomized Trials. Crit. Care Med..

[149] Font M.D., Thyagarajan B., Khanna A.K. (2020). Sepsis and Septic Shock—Basics of diagnosis, pathophysiology and clinical decision making. Med. Clin. N. Am..

[150] Cioccari L., Jakob S.M., Takala J. (2021). Should Vasopressors Be Started Early in Septic Shock?. Semin. Respir. Crit. Care Med..

[151] Ospina-Tascón G.A., Hernandez G., Alvarez I., Calderón-Tapia L.E., Manzano-Nunez R., Sánchez-Ortiz A.I., Quiñones E., Ruiz-Yucuma J.E., Aldana J.L., Teboul J.L. (2020). Effects of very early start of norepinephrine in patients with septic shock: A propensity score-based analysis. Crit. Care.

[152] Chalfin D.B. (2022). Vasopressor Therapy Early, or Vasopressors Later? Still an Important Question in Septic Shock. Crit. Care Med..

[153] Yeo H.J., Lee Y.S., Kim T.H., Jang J.H., Lee H.B., Oh D.K., Park M.H., Lim C.M., Cho W.H., Korean Sepsis Alliance (KSA) Investigators (2022). Vasopressor Initiation Within 1 Hour of Fluid Loading Is Associated with Increased Mortality in Septic Shock Patients: Analysis of National Registry Data. Crit. Care Med..

[154] O’Driscoll B.R., Howard L.S., Earis J., Mak V. (2017). British Thoracic Society Guideline for oxygen use in adults in healthcare and emergency settings. BMJ Open Respir. Res..

[155] Stolmeijer R., Bouma H.R., Zijlstra J.G., Drost-de Klerck A.M., Ter Maaten J.C., Ligtenberg J.J.M. (2018). A Systematic Review of the Effects of Hyperoxia in Acutely Ill Patients: Should We Aim for Less?. Biomed. Res. Int..

[156] Barbateskovic M., Schjørring O.L., Krauss S.R., Meyhoff C.S., Jakobsen J.C., Rasmussen B.S., Perner A., Wetterslev J. (2021). Higher vs Lower Oxygenation Strategies in Acutely Ill Adults: A Systematic Review with Meta-Analysis and Trial Sequential Analysis. Chest.

[157] De Monnin K., Terian E., Yaegar L.H., Pappal R.D., Mohr N.M., Roberts B.W., Kollef M.H., Palmer C.M., Ablordeppey E., Fuller B.M. (2022). Low Tidal Volume Ventilation for Emergency Department Patients: A Systematic Review and Meta-Analysis on Practice Patterns and Clinical Impact. Crit. Care Med..

[158] Gottlieb M., Chesis M., Long B. (2022). What is the Impact of Low Tidal Volume Ventilation for Emergency Department Patients?. Ann. Emerg. Med..

[159] MacIntyre N.R. (2019). Physiologic Effects of Noninvasive Ventilation. Respir. Care.

[160] Frat J.P., Thille A.W., Mercat A., Girault C., Ragot S., Perbet S., Prat G., Boulain T., Morawiec E., Cottereau A. (2015). High-flow oxygen through nasal cannula in acute hypoxemic respiratory failure. N. Engl. J. Med..

[161] Mauri T., Turrini C., Eronia N., Grasselli G., Volta C.A., Bellani G., Pesenti A. (2017). Physiologic Effects of High-Flow Nasal Cannula in Acute Hypoxemic Respiratory Failure. Am. J. Respir. Crit. Care Med..

[162] Xuan L., Ma J., Tao J., Zhu L., Lin S., Chen S., Pan S., Zhu D., Yi L., Zheng Y. (2021). Comparative study of high flow nasal catheter device and noninvasive positive pressure ventilation for sequential treatment in sepsis patients after weaning from mechanical ventilation in intensive care unit. Ann. Palliat. Med..

[163] Tongyoo S., Tantibundit P., Daorattanachai K., Viarasilpa T., Permpikul C., Udompanturak S. (2021). High-flow nasal oxygen cannula vs. noninvasive mechanical ventilation to prevent reintubation in sepsis: A randomized controlled trial. Ann. Intensive Care.

[164] Kim E., Jeon K., Oh D.K., Cho Y.J., Hong S.B., Lee Y.J., Lee S.M., Suh G.Y., Park M.H., Lim C.M. (2021). Failure of High-Flow Nasal Cannula Therapy in Pneumonia and Non-Pneumonia Sepsis Patients: A Prospective Cohort Study. J. Clin. Med..

[165] Iba T., Levi M., Levy J.H. (2020). Sepsis-Induced Coagulopathy and Disseminated Intravascular Coagulation. Semin. Thromb. Hemost..

[166] Iba T., Levy J.H., Warkentin T.E., Thachil J., van der Poll T., Levi M., Scientific and Standardization Committee on DIC, and the Scientific and Standardization Committee on Perioperative and Critical Care of the International Society on Thrombosis and Haemostasis (2019). Diagnosis and management of sepsis-induced coagulopathy and disseminated intravascular coagulation. J. Thromb. Haemost..

[167] Li X., Ma X. (2017). The role of heparin in sepsis: Much more than just an anticoagulant. Br. J. Haematol..

[168] Zhang X., Li X. (2022). The Role of Histones and Heparin in Sepsis: A Review. J. Intensive Care Med..

[169] Liu Z., Zhu H., Ma X. (2014). Heparin for treatment of sepsis: A systemic review. Zhonghua Wei Zhong Bing Ji Jiu Yi Xue.

[170] Yini S., Heng Z., Xin A., Xiaochun M. (2015). Effect of unfractionated heparin on endothelial glycocalyx in a septic shock model. Acta Anaesthesiol. Scand..

[171] Arabi Y.M., Al-Hameed F., Burns K.E.A., Mehta S., Alsolamy S.J., Alshahrani M.S., Mandourah Y., Almekhlafi G.A., Almaani M., Al Bshabshe A. (2019). Adjunctive Intermittent Pneumatic Compression for Venous Thromboprophylaxis. N. Engl. J. Med..

[172] Aleman L., Guerrero J. (2018). Hiperglicemia por sepsis: Del mecanismo a la clínica [Sepsis hyperglycemia in the ICU: From the mechanism to the clinic]. Rev. Med. Chil..

[173] Rivas A.M., Nugent K. (2021). Hyperglycemia, Insulin, and Insulin Resistance in Sepsis. Am. J. Med. Sci..

[174] Zohar Y., Zilberman Itskovich S., Koren S., Zaidenstein R., Marchaim D., Koren R. (2021). The association of diabetes and hyperglycemia with sepsis outcomes: A population-based cohort analysis. Intern. Emerg. Med..

[175] See K.C. (2021). Glycemic targets in critically ill adults: A mini-review. World J. Diabetes.

[176] Jeger R.V., Seeberger M.D., Keller U., Pfisterer M.E., Filipovic M. (2007). Oral hypoglycemics: Increased postoperative mortality in coronary risk patients. Cardiology.

[177] Rim J., Gallini J., Jasien C., Cui X., Phillips L., Trammell A., Sadikot R.T. (2022). Use of oral anti-diabetic drugs and risk of hospital and intensive care unit admissions for infections. Am. J. Med. Sci..

[178] Fujishima S., Gando S., Saitoh D., Kushimoto S., Ogura H., Abe T., Shiraishi A., Mayumi T., Sasaki J., Kotani J. (2021). Incidence and Impact of Dysglycemia in Patients with Sepsis Under Moderate Glycemic Control. Shock.

[179] Granholm A., Zeng L., Dionne J.C., Perner A., Marker S., Krag M., MacLaren R., Ye Z., Møller M.H., Alhazzani W. (2019). Predictors of gastrointestinal bleeding in adult ICU patients: A systematic review and meta-analysis. Intensive Care Med..

[180] Krag M., Perner A., Møller M.H. (2016). Stress ulcer prophylaxis in the intensive care unit. Curr Opin Crit. Care.

[181] Huang M., Han M., Han W., Kuang L. (2021). Proton pump inhibitors versus histamine-2 receptor blockers for stress ulcer prophylaxis in patients with sepsis: A retrospective cohort study. J. Int. Med. Res..

[182] D’Silva K.M., Mehta R., Mitchell M., Lee T.C., Singhal V., Wilson M.G., McDonald E.G. (2021). Proton pump inhibitor use and risk for recurrent Clostridioides difficile infection: A systematic review and meta-analysis. Clin. Microbiol. Infect..

[183] Khwaja A. (2012). KDIGO clinical practice guidelines for acute kidney injury. Nephron Clin. Pract..

[184] Bagshaw S.M., George C., Bellomo R., ANZICS Database Management Committee (2008). Early acute kidney injury and sepsis: A multicentre evaluation. Crit. Care.

[185] Bagshaw S.M., Lapinsky S., Dial S., Arabi Y., Dodek P., Wood G., Ellis P., Guzman J., Marshall J., Parrillo J.E. (2009). Acute kidney injury in septic shock: Clinical outcomes and impact of duration of hypotension prior to initiation of antimicrobial therapy. Intensive Care Med..

[186] Cobussen M., Verhave J.C., Buijs J., Stassen P.M. (2023). The incidence and outcome of AKI in patients with sepsis in the emergency department applying different definitions of AKI and sepsis. Int. Urol. Nephrol..

[187] Hellman T., Uusalo P., Järvisalo M.J. (2021). Renal Replacement Techniques in Septic Shock. Int. J. Mol. Sci..

[188] Zarbock A., Kellum J.A., Schmidt C., Van Aken H., Wempe C., Pavenstädt H., Boanta A., Gerß J., Meersch M. (2016). Effect of Early vs Delayed Initiation of Renal Replacement Therapy on Mortality in Critically Ill Patients with Acute Kidney Injury: The ELAIN Randomized Clinical Trial. JAMA.

[189] Pasin L., Boraso S., Tiberio I. (2019). Early initiation of renal replacement therapy in critically ill patients: A meta-analysis of randomized clinical trials. BMC Anesthesiol..

[190] Barbar S.D., Clere-Jehl R., Bourredjem A., Hernu R., Montini F., Bruyère R., Lebert C., Bohé J., Badie J., Eraldi J.P. (2018). Timing of Renal-Replacement Therapy in Patients with Acute Kidney Injury and Sepsis. N. Engl. J. Med..

[191] Chen W.Y., Cai L.H., Zhang Z.H., Tao L.L., Wen Y.C., Li Z.B., Li L., Ling Y., Li J.W., Xing R. (2021). The timing of continuous renal replacement therapy initiation in sepsis-associated acute kidney injury in the intensive care unit: The CRTSAKI Study (Continuous RRT Timing in Sepsis-associated AKI in ICU): Study protocol for a multicentre, randomised controlled trial. BMJ Open.

[192] Annane D., Renault A., Brun-Buisson C., Megarbane B., Quenot J.P., Siami S., Cariou A., Forceville X., Schwebel C., Martin C. (2018). Hydrocortisone plus Fludrocortisone for Adults with Septic Shock. N. Engl. J. Med..

[193] Annane D., Bellissant E., Bollaert P.E., Briegel J., Keh D., Kupfer Y., Pirracchio R., Rochwerg B. (2019). Corticosteroids for treating sepsis in children and adults. Cochrane Database Syst. Rev..

[194] Fang F., Zhang Y., Tang J., Lunsford L.D., Li T., Tang R., He J., Xu P., Faramand A., Xu J. (2019). Association of Corticosteroid Treatment with Outcomes in Adult Patients with Sepsis: A Systematic Review and Meta-analysis. JAMA Intern. Med..

[195] Keh D., Trips E., Marx G., Wirtz S.P., Abduljawwad E., Bercker S., Bogatsch H., Briegel J., Engel C., Gerlach H. (2016). Effect of Hydrocortisone on Development of Shock Among Patients with Severe Sepsis: The HYPRESS Randomized Clinical Trial. JAMA.

[196] Zhao Y., Ding C. (2018). Effects of Hydrocortisone on Regulating Inflammation, Hemodynamic Stability, and Preventing Shock in Severe Sepsis Patients. Med. Sci. Monit..

[197] Fong K.M., Au S.Y., Ng G.W.Y. (2021). Steroid, ascorbic acid, and thiamine in adults with sepsis and septic shock: A systematic review and component network meta-analysis. Sci. Rep..

[198] Jacobi J. (2002). Pathophysiology of sepsis. Am. J. Health Syst. Pharm..

[199] Maciel A.T., Noritomi D.T., Park M. (2010). Metabolic acidosis in sepsis. Endocr. Metab. Immune Disord. Drug Targets.

[200] Suetrong B., Walley K.R. (2016). Lactic Acidosis in Sepsis: It’s Not All Anaerobic: Implications for Diagnosis and Management. Chest.

[201] Rudnick M.R., Blair G.J., Kuschner W.G., Barr J. (2020). Lactic Acidosis and the Role of Sodium Bicarbonate: A Narrative Opinion. Shock.

[202] Yagi K., Fujii T. (2021). Management of acute metabolic acidosis in the ICU: Sodium bicarbonate and renal replacement therapy. Crit. Care.

[203] Zhang Z., Zhu C., Mo L., Hong Y. (2018). Effectiveness of sodium bicarbonate infusion on mortality in septic patients with metabolic acidosis. Intensive Care Med..

[204] Jung B., Rimmele T., Le Goff C., Chanques G., Corne P., Jonquet O., Muller L., Lefrant J.Y., Guervilly C., Papazian L. (2011). Severe metabolic or mixed acidemia on intensive care unit admission: Incidence, prognosis and administration of buffer therapy. A prospective, multiple-center study. Crit. Care.

[205] Duhon B., Attridge R.L., Franco-Martinez A.C., Maxwell P.R., Hughes D.W. (2013). Intravenous sodium bicarbonate therapy in severely acidotic diabetic ketoacidosis. Ann. Pharmacother..

[206] Jaber S., Paugam C., Futier E., Lefrant J.Y., Lasocki S., Lescot T., Pottecher J., Demoule A., Ferrandière M., Asehnoune K. (2018). Sodium bicarbonate therapy for patients with severe metabolic acidaemia in the intensive care unit (BICAR-ICU): A multicentre, open-label, randomised controlled, phase 3 trial. Lancet.

[207] Inage S., Yajima R., Nagahara S., Kazama A., Takamura M., Shoji T., Kadoi M., Tashiro Y., Ise Y. (2022). Acetaminophen-induced hypotension in sepsis. J. Pharm. Health Care Sci..

[208] Sakkat A., Alquraini M., Aljazeeri J., Farooqi M.A.M., Alshamsi F., Alhazzani W. (2021). Temperature control in critically ill patients with fever: A meta-analysis of randomized controlled trials. J. Crit. Care.

